# Progress in Surgery

**Published:** 1897-01-23

**Authors:** 


### Progress in Surgery.

### SURGERY OF THE LIVER AND GALL BLADDER.

**Hydatid Cysts of the Liver** is the subject of a paper by Chauffard,[1] who has had a fatal case following on puncture of the cyst for diagnostic purposes. Every antiseptic precaution was observed, and no pain was complained of. A few moments later the patient felt suddenly ill, intense irritation of the skin was experienced, followed by two severe epileptiform seizures, and death ensued from cardiac collapse twenty-five minutes after operation. Post-mortem evidence was negative except as regards the liver. Here the trabecular arrangement was disturbed, the glandular element having obliterated the capillary network, and leaving an almost entirely cellular parenchyma. Death after puncture usually results in cases of intra-peritoneal rupture. Two theories only can account for this case: (1) That the symptoms were reflex, and of traumatic origin; (2) that they were due to intoxication by a special hydatid poison. The latter is the generally accepted, and in this case the most likely, view. The three groups of symptoms—cutaneous, cerebro-spinal, and myocardiac—which are frequently met with after puncture of hydatid cysts, can scarcely be due to an ordinary traumatic reflex; there must be some reflex connected with the puncture of the cyst itself. In the mildest form of hydatid intoxication—urticaria—patients are differently affected, and individual idiosyncrasy, with regard to the poison resulting from effusion into the peritoneum of a few drops of the fluid, is the only way of accounting for the fatal effects produced in this case. Bolognesi[2] says the best surgical treatment of hydatid cysts is direct incision by the anterior abdominal route, by the transpleural route, or by the lumbar incision. Median or lateral laparotomy should be reserved for anterior cysts. The transpleural route, with resection of the ribs, is the best way of reaching sub-diaphragmatic cysts which are deeply placed. In this method two or three inches of the median portion of the seventh, eighth, or ninth ribs may be excised. The thoracic wall must be firmly pressed upon while the pleura is being cut through. The diaphragm is then incised, and the borders of this incision are held out until the two pleural surfaces are sutured together. The cyst is then evacuated and drained. Lumbar incision allows the surgeon to reach cysts in the posterior and lower part of the liver. Incision, combined with partial excision of the walls, is only practicable in the treatment of cysts reached by laparotomy. Delbet[3] has treated certain hydatid cysts by an operation which he describes as "upholstering." When the cystic cavity, after having been emptied and cleansed, is found to be so closely adherent to the liver as to prevent its being excised, "upholstering" is resorted to, the walls of the cavity being stitched together with two coarse catgut sutures. The incision in the liver is then closed by a continuous catgut suture, and the organ returned into the abdominal cavity without drainage. The author restricts this operation to hydatid cysts with clear, non-infected contents; and, in case there is communication with the bile ducts, this method should only be resorted to if the bile is not infected.

**Hepatic Abscess,** says Ricard,[4] though often sterile, is not always so. In acute abscesses due to general streptococcus infection the virulence of the pus is very intense, and indicates prompt intervention. The cases in which the pus is usually sterile are those of slowly-developed abscess following chronic dysentery. When situated, as is often the case, at the upper and back part of the right lobe, they are best treated by resection of a portion of the ninth or tenth rib, after the manner described above for hydatid cysts. When the anterior portion of the liver is involved, the abscess should be exposed by anterior laparotomy, the edges of the external wound being stitched to the surface of the liver if possible. The author is opposed to the practice of scraping the inner surface of the abscess cavity, and holds that simple injections after incision are quite sufficient and less dangerous. Bramwell and Stiles[5] report a case treated by the transpleural route. The latter considers that, in the absence of local signs, the seventh interspace should be first selected in making an exploratory puncture. When there is no downward enlargement of the liver, puncture through the ninth space is too low, and might wound the colon or gall-bladder. The advantage of making a bacteriological examination of the pus before proceeding to operate is that, in the event of its being found sterile, there is no necessity to perform the operation in two stages, and the patient is thus spared the anxiety and shock of a second operation. In shutting off the pleural cavity there is a double advantage in passing the sutures before incising the pleura: (1) That they are more easily and satisfactorily introduced; and (2) that very little air enters the chest. Further, in case the liver should not have become adherent to the diaphragm, the sutures should be carried through the latter into the substance of the liver.

**Movable Liver** is a rare affection. Bolognesi[6] says there is no indication for intervention if it can be held in place by an apparatus. If the gall bladder is distended, cholecystotomy is indicated, this being sufficient to cause fixation of the enlarged and floating lobule. The entire organ, or one lobe only, may be displaced. Langerbuch has twice performed a partial hepatopexy for movable liver. The operation for fixing the liver completely is performed by making an incision parallel to the costal cartilages of the right side, along the outer borber of the right rectus muscle. The liver having been placed in proper position and held there, four silk threads are passed through it two or three centimetres from its sharp anterior edge, their lower ends being brought through the lower border of the abdominal incision, and their upper ends carried through the costal cartilages and the overlying tissues. The threads are then knotted, and the abdomen is closed.

**Cholelithiasis.**—Dunn[7] thinks that if the attacks of colic are frequent, the calculi not passed, and the symptoms not relieved by reasonable medical treatment of a few weeks' duration, it is safer to perform cholecystotomy than to allow the patient to run the risk of cholangeitis and duct impaction. In 95 per cent. of cases gall stones occupy the gall bladder alone, or it and the cystic duct alone. In the vast majority of cases, therefore, the indication appears to be to open the gall bladder, remove the stones and *débris*, and temporarily drain the diseased organ. Various conditions in connection with the remaining minority will demand a modification of the operation. Choledochotomy may be necessary, but it is always difficult, frequently dangerous, and sometimes impossible. Ferguson[8] says we must open the gall bladder if the patient has colic and jaundice, or if the gall bladder is so enlarged as to form an apparent tumour. Jaundice cases stand operation and chloroform badly, and therefore as soon as we see bile our work is finished. There are two ways of getting at the common duct—one internal and the other external to the duodenum, of which the former is the better. In order to tell whether or not the common duct is patulous, a good way is to take a glass tube that is bulbous at one end, with a rubber bulb attached to the other. This will become held in the duct. If water will then flow through the bulb it is well to stop operating. As regards diagnosis, Taffier[9] says there are two groups of cases—those in which an enlargement of the gall bladder can be felt, and those in which no tumour can be detected. In the latter there is nothing to go by, except the previous history of the patient and the existence of pain over the gall bladder. Pain, however, may be localised at a considerable distance, and thus be mistaken for such diseases as appendicitis. When there is a tumour it may be connected with the liver or kidney, though a differential diagnosis can generally be made by palpation alone. In one case of supposed enlargement of the gall bladder, the tumour proved to be an old-standing extra-uterine fœtation. Michaux[10] says he prefers cholecystectomy to cholecystostomy; it is more radical, gets rid of a gall bladder which is infected, and is not more serious; for of twelve patients subjected to cholecystectomy, in two of whom this operation was combined with choledochotomy, he had only three deaths. Broca[11] also agrees that cholecystectomy is generally to be preferred to cholecystostomy in the treatment of biliary lithiasis. Rontier[12] also agrees with these observers, but Monod[13] maintains that cholecystostomy is infinitely less grave. Roper[14] describes an interesting case of gall-stones which resembled the symptoms of intussusception. Kehr's[15] mortality in general gall-bladder surgery is eight per cent. of all cases, and only one per cent. considering merely uncomplicated cases—a result which has never before been equalled. Of thirty cases of choledochotomy he had only two deaths, whereas the mortality is generally about thirty per cent. Körte[16] calls attention to the fact, of which he has obtained ample proof by bacteriological research, that the contents of the gall bladder are often found to be infected, without becoming purulent. Vautrin[17] gives an account of the various operations for calculous obstruction of the common duct: (1) Pressing the calculus into the duodenum or into the gall bladder, from which it is removed by cholecystostomy. This, though the ideal treatment, is seldom practicable. Breaking the stone and pressing the fragments into the gall bladder is unsatisfactory, because some of them may escape into the hepatic duct, and give rise to recurrence. (2) Crushing calculus *in situ*, and compressing fragments into duodenum. This should not be done unless calculi can be crushed with the fingers. Needling calculi is dangerous. (3) Choledochotomy: In twenty-seven cases sixteen were fatal. When there are calculi in the gall bladder a further operation is necessary. Cholecystostomy is best, and cholecystectomy should only be done when the gall bladder is very friable or much inflamed. (4) Duodenotomy: This might occasionally be preferable to choledochotomy if the stone has impacted in the diverticulum Vateri. (5) In an exploratory operation, where it is impossible or inadvisable to perform choledochotomy, it is best to perform cholecystostomy, and save the patient from the results of cholæmia. (6) In the other palliative operation of cholecystenterostomy there is danger of infection from the intestine. Yversen has brought the dilated bile duct to the surface of the body—choledochostomy. Halsted[18] says it is not always easy to explore the cystic duct, but one can explore the common duct. It can be done by cutting through the mesocolon and drawing the omentum up and the duodenum out, when the common duct will be seen. He prefers the vertical incision as giving more room. An osteoplastic operation in the ribs may be necessary. He thinks that operation in two stages and cholecystenterostomy will soon be abandoned. Michaux[19] prefers a lateral to a median incision, for it enables one to deal with the gall bladder and common duct at the same time, and this is often necessary. Quénu,[20] however, prefers the median incision for operations on the ductus choledochus, because it is easier to make than a lateral incision, and gives rise to less hæmorrhage. Ricard[21] is of the same opinion. Kelynack[22] records a rare case of primary malignant growth of the gall bladder, associated with cholelithiasis and secondary cancerous deposits in the liver. Compression of the transverse colon and intestinal obstruction ensued, for which a colotomy was performed, but the patient died on the following day.

[1] Brit. Med. Journ., Aug. 29th, 1896, and Sem. Méd., Aug. 8th, 1896. [2] Therap. Gazette, July 15th, 1896. [3] Med. Week, May 29th, 1896. [4] Brit. Med. Journ., June 6th, 1896, and Bulletin de la Soc. de Chirurg., 1-2, 1896. [5] Edin. Med. Journ., October, 1896, and Brit. Med. Journ., July 11th, 1896. [6] Therap. Gazette, July 15th, 1896. [7] Med. Rec., New York, May 23rd, 1896. [8] Ibidem, p. 739. [9] Med. Week, June 5th, 1896. [10] Ibidem, May 15th, 1896. [11] Ibidem, May, 15th 1896. [12] Ibidem, May 29th, 1896. [13] Ibidem, May 29th, 1896, [14] Lancet, Aug. 22nd, 1896, [15] Med. Week, June 12th, 1896. [16] Ibidem. [17] Brit. Med. Journ., Aug. 22nd, 1896, and Rev. de Chir., June 10th, 1896. [18] Med. Rec., New York, May 16th, 1896. [19] Med. Week, July 10th, 1896. [20] Ibidem, July 3rd, 1396. [21] Ibidem, June 20th, 1896. [23] Med. Press and Circ., Sept. 9th, 1896.

